# Identifying preoperative radiographic metrics to guide surgical selection in lumbar spondylolisthesis and stenosis

**DOI:** 10.1016/j.xnsj.2025.100784

**Published:** 2025-08-21

**Authors:** John A. Hipp, Bradford L. Currier, Trevor F. Grieco, Job L.C. Van Susante

**Affiliations:** a Medical Metrics, Inc., 2000 Bering, Suite 800, Houston, TX 77057, United States; b Department of Orthopedics, Mayo Clinic, 565 1st St SW, Rochester, MN 55902, United States; c Department of Orthopedics, Rijnstate Hospital, Wagnerlaan 55, 6815 AG Arnhem, The Netherlands

**Keywords:** Lumbar, Spondylolisthesis, Stenosis, Surgical selection, Fusion, Decompression, Threshold-limit graphical approach, Predictive metrics, Radiographic, Spinal instability

## Abstract

**Background Context:**

“Instability” often drives the decision to add fusion to decompression, yet most instability criteria lean solely on sagittal translation and have never been rigorously validated. The potential of a metric for sagittal plane translation to help decide whether fusion should be added to decompression surgery for symptomatic lumbar stenosis with spondylolisthesis was recently reported. Building on imaging and outcomes from that study, we investigated whether other motion metrics may help to predict postoperative disability and patient‐reported outcomes in lumbar stenosis with spondylolisthesis.

**Methods:**

Radiographic metrics were retrospectively calculated from the prospectively collected flexion-extension radiographs of 61 patients with lumbar spinal stenosis and spondylolisthesis. A threshold-limit graphical approach was used to identify metrics and thresholds predictive of the Oswestry Disability Index, leg/buttock pain, and patient satisfaction. Outcomes were compared across groups defined by these threshold levels using statistical analysis.

**Results:**

Decompression-only surgery was associated with poorer outcomes in patients exhibiting vertical instability or significant spondylolisthesis changes between flexion and extension. Conversely, decompression-plus-fusion surgery yielded worse outcomes in cases without substantial dynamic spondylolisthesis.

**Conclusions:**

A broader definition of spinal instability may be needed when deciding whether to include fusion in treating lumbar stenosis with spondylolisthesis. Preoperative vertical instability and dynamic slip may be important in addition to translational instability. Larger prospective studies are warranted, but these metrics could help guide the decision on whether fusion is necessary and likely to improve outcomes for a common spinal disorder.

## Introduction

Determining whether fusion should be added to decompression in patients with spondylolisthesis and stenosis remains a clinical challenge [1,2]. A recent evidence-based review found contradictory conclusions regarding the efficacy of decompression plus fusion versus decompression alone [2]. That review concluded: “Surgeons should closely review pre-operative imaging for signs of instability in order to better identify appropriate patients for each indication.” Surgeons often use spinal instability as a key factor when deciding whether to perform decompression surgery alone or to add fusion [3]. However, this practice is questionable because there are no well-validated tests for instability [[4], [5], [6], [7]]. Traditional thresholds for intervertebral rotation (eg, >10° or >15°) or translation (eg, >4.5mm) lack validation against a gold standard and are frequently applied outside their original context of use in a trauma setting [8,9]. Furthermore, conventional flexion-extension radiographs depend heavily on patient effort, which can be inconsistent due to pain, fear avoidance, or muscle spasms [[10], [11], [12]]. If the spine is not sufficiently stressed, this can potentially lead to false negative instability diagnoses. Consequently, more robust and reliable instability metrics are needed to improve surgical decision-making.

A recent study investigated the potential of a metric for sagittal plane translational intervertebral motion, corrected for the amount of rotation, to help decide whether fusion should be added to decompression surgery for symptomatic lumbar stenosis with spondylolisthesis [13]. In that prospective study (ClinicalTrials.gov ID NCT03754972), surgeons first chose a surgical plan—either decompression-only or decompression plus fusion—before receiving a standardized measure of translational instability. If the measure was greater than 2, indicating instability, surgeons who originally planned decompression alone considered adding fusion. If it was less than 2, surgeons who planned decompression plus fusion considered switching to decompression-only. Surgeons were not required to change their plans and chose not to do so in 8% of cases. Overall, 29% of the original plans were revised, decreasing the decompression-plus-fusion surgeries from 32% to 21%.

Using 1-year outcomes from the prospective study, we hypothesize that radiographic metrics beyond translational instability may also predict clinical results.

## Methods

### Patients and surgeries

The inclusion and exclusion criteria and the IRB approval of the prospective study have been described previously (https://clinicaltr- ials.govstudy/NCT03754972) [13]. Briefly, all patients had single-level lumbar spinal stenosis and spondylolisthesis with symptoms consistent with these findings. Informed consent was obtained for all participants in the original prospective study.

Since the type of decompression can influence lumbar stability, the following summarizes the operative techniques [14]. Interlaminar decompression was performed through a posterior midline approach. Central and foraminal decompression was achieved by partial laminectomy in combination with bilateral medial facetectomies up to the medial pedicle wall, and into the lateral recess. In case of additional fusion, a standard pedicle screw-rod fusion instrumentation was performed along with interbody fusion. The disc space and endplates were cleaned, followed by a standard titanium (TLIF) cage implantation. Locally harvested bone from the decompression was impacted behind the cage to promote interbody fusion. The wound was closed in layers after all surgeries, followed by standard postoperative care.

### Flexion-extension radiographs and automated analysis

Flexion-extension radiographs were obtained using a protocol intended to encourage a minimum of 5 degrees of intervertebral rotation at every level from L1-L2 to L5-S1. Technicians were trained on this protocol (https://www.youtube.com/watch?v=YDcMMZdc7dc), and patients watched a training video before the exam (https://youtu.be/XZyKkv3zO20). Using fully automated and FDA-cleared technology (SpineCAMP^TM^, Medical Metrics, Inc. Houston, TX), multiple intervertebral motion and alignment metrics were calculated from the preoperative flexion-extension radiographs, providing results equivalent to previously validated semi-automated Quantitative Motion Analysis (QMA®, Medical Metrics Inc, Houston, TX) technology [15,16].

### Intervertebral motion metrics

**Fig. 1:**
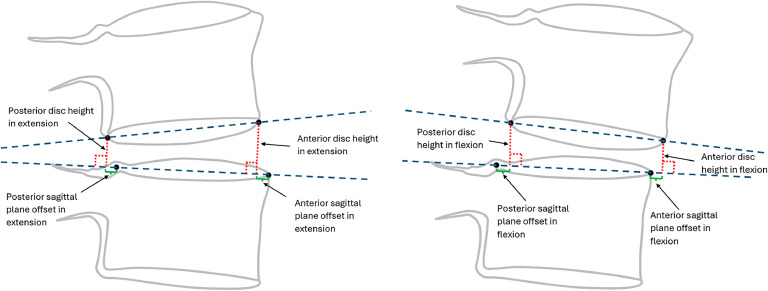
Diagram illustrating measurements of anterior and posterior disc heights, which were used in calculating the vertical instability index [7] and in calculating the disc height index [18]. This diagram also illustrates the measurement of anterior and posterior sagittal plane offsets in flexion and extension. The sagittal plane offsets are used in calculating the spondylolisthesis index and the change in spondylolisthesis index.
• Disc Height Index (DHI): This index quantifies disc height as the average of 4 disc heights: anterior and posterior, in flexion and extension. This average was reported relative to the disc heights of healthy discs [18]. It accounts for the variability between levels.
• Spondylolisthesis Index (SI): This index quantifies the sagittal plane offset (Fig. 1) relative to asymptomatic volunteers while accounting for variations in disc area, endplate width ratio (EPWR), disc height, and spinal level [18]. The change in the SI between flexion and extension was also investigated.

All intervertebral metrics have been previously described in detail and were reported relative to the mean for radiographically normal levels [[7], [17], [18]]. All these metrics are within normal limits when they are between −2 and +2 and are expressed in units of standard error of the forecast (SEF). The SEF provides a point estimate of the variability found in radiographically normal discs for the amount of rotation. A value of +3 indicates that the metric is 3 SEF above the average for asymptomatic volunteers. The metrics studied included the following:

• Translational instability index (TI-Index): This index measures the sagittal plane translation of the superior vertebra's posterior-inferior corner relative to the inferior vertebra's posterior-superior corner [7]. This metric is level specific and accounts for variability between individuals in the size of vertebrae, and sagittal plane rotation between vertebrae (due to variability in patient effort when asked to flex and extend).
• Anterior and posterior vertical instability index (AVI-Index, PVI-Index): These metrics quantify the amount of disc height change between flexion and extension (Fig. 1), measured at the anterior- and posterior-most aspects of the disc space [7]. They account for patient variability in the amount of rotation and are level-specific. Examples of levels with an abnormal AVI-Index can be found online at: https://qims.amegroups.org/article/view/135403/html.

Intervertebral translation was also measured in millimeters. Because no radiographic scaling marker was available, an assumed anteroposterior width was assigned to the superior endplate of each inferior vertebra based on 870 lumbar X-rays from a quality-control (QC) database (Medical Metrics, Inc., Houston, TX). These QC X-rays included a scaling ring placed in the mid-sagittal plane, which allowed for the accurate calculation of an image scale factor. From these QC data, average anteroposterior widths for the superior endplate of each vertebra level were established. Translation exceeding 4mm was used since it is a commonly cited criterion for instability [19,20].

### Identification of metrics and thresholds

The preoperative radiographic metrics and 12-month outcomes data for 48 decompression-only and 14 decompression plus fusion patients were retrospectively analyzed using a threshold-limit (TL) graphical method [21]. The TL graphical method is a simple way to visualize associations between preoperative metrics and outcomes and to identify threshold values of the metric that can help predict particularly good or bad outcomes [21]. (https://youtu.be/z8DSUeTv-Zg) For example, a threshold limit graph can plot the mean value of an outcome variable (y-axis) against the varying threshold levels of a predictive metric (x-axis). For each threshold on the x-axis, the mean outcome is calculated using only the data where the predictive metric exceeded (or was below) the threshold.

The threshold-limit method helps identify the threshold level at which an outcome substantially deviates from the 95% confidence interval (CI) derived from all data, with a clear upward or downward trajectory out of the 95% CI. If a metric does not affect an outcome, no clear pattern will be observed in the TL graph and the line will not pass out of the 95% confidence interval. Once a threshold is identified, statistical tests such as 1-way analysis of variance can determine whether there is a significant difference in outcomes when data are grouped based on the predictive variable being above or below the chosen threshold. TL graphs can also plot the proportion of patients achieving a goal, such as being satisfied with the surgery, on the y-axis against the varying threshold levels of a predictive metric on the x-axis.

The TL graphical approach was implemented using Python [22] and various Python libraries. The code is freely available. (GitHub - drjah/Threshold-Limit-Graphs: Python code to create threshold limit graphs) The TL graphs are not intended to provide clinical guidance by themselves. They are intended to be used to discover potential predictive metrics that could be investigated in future prospective clinical trials, along with the threshold levels for each metric that might prove effective using conventional statistical tests.

Implementation of the TL graphical method requires first creating an Excel format file specifying:

• Outcomes to predict.
• Preoperative metrics that are hypothesized to help predict the outcome.
• Data filters that need to be applied to the complete data set before analyzing the data, for example, only use data for the treatment level in decompression-only patients.

These are all specified in an input file, and then TL graphs are generated and interpreted. Interpretation involves identifying apparent trends in the data (eg consistently worsening or improving outcomes as the threshold level of a predictive variable changes), and identifying when a trend rises above or falls below the 95% confidence interval (CI) established using the complete dataset. A shaded rectangle shows the 95% CI in each graph. Appendix 1 lists the selected outcomes, potential predictive metrics, and the filters applied to the data. After identifying potential thresholds, supplemental analysis of variance tests and tests of proportions were completed using Stata (ver 15, College Station, TX, USA).

### Clinical outcomes

The 12-month outcomes were the Oswestry Disability Index (ODI) [23], leg/buttock pain (“How severe was your leg and/or buttock pain for the last week?") measured on a numeric rating scale, and patient satisfaction. The patients recorded their satisfaction as satisfied, uncertain, or dissatisfied. For data analysis purposes, this was recoded as 1 = satisfied or 0 = uncertain or dissatisfied. The ODI was analyzed based on the averages for each subgroup and the proportion of patients with an ODI improvement of >20 points [24].

## Results

All patients were diagnosed with single-level degenerative spondylolisthesis with stenosis, and had single-level surgery. Of the 61 patients with complete data (48 decompression-only, 13 decompression-plus-fusion), none showed >4 mm sagittal plane intervertebral translation, and only 3 exceeded 3.5 mm preoperatively. A high TI-Index was not significantly associated with 1-year outcomes in either surgical group. This is as expected, given that an equivalent measure of translational instability was used to determine what type of surgery was performed. The DHI was also not significantly associated with outcomes in either surgical group.

**Fig. 2:**
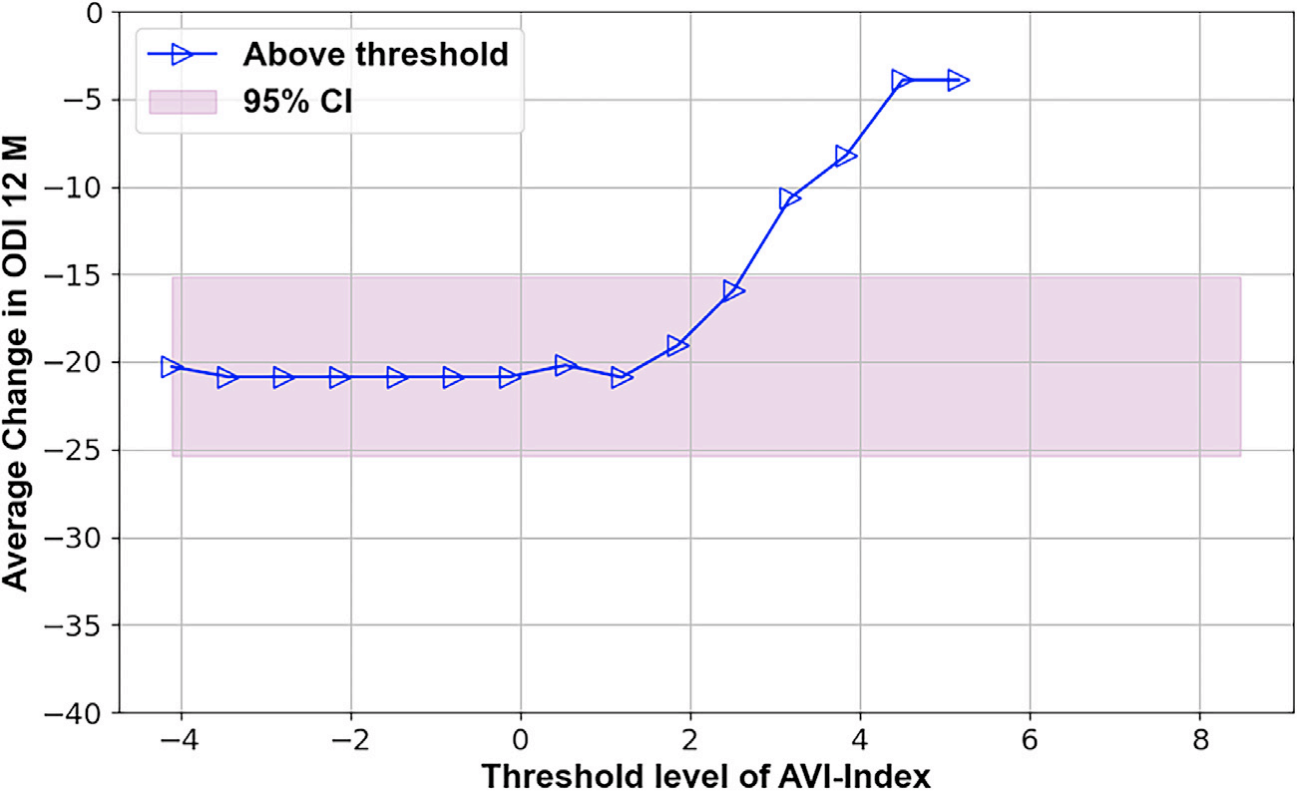
TL graph showing how the anterior vertical instability index (AVI-Index) may predict changes in ODI scores 1 year after decompression-only surgery for degenerative spondylolisthesis.

**Fig. 3:**
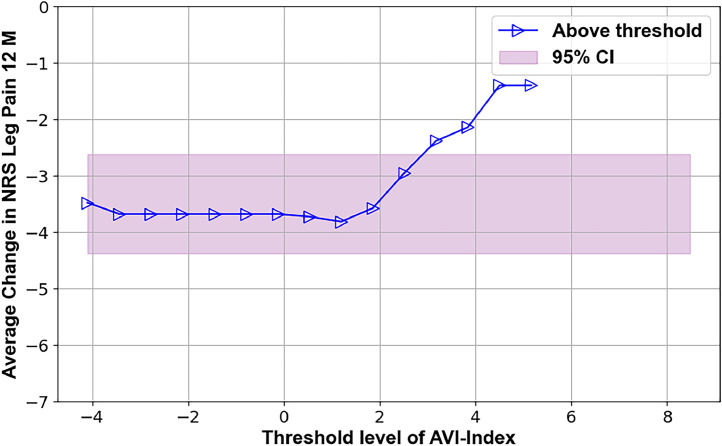
TL graph indicating the potential of the anterior vertical instability index to predict the change in leg/buttock pain scores 1 year following decompression-only surgery for degenerative spondylolisthesis.

**Fig. 4:**
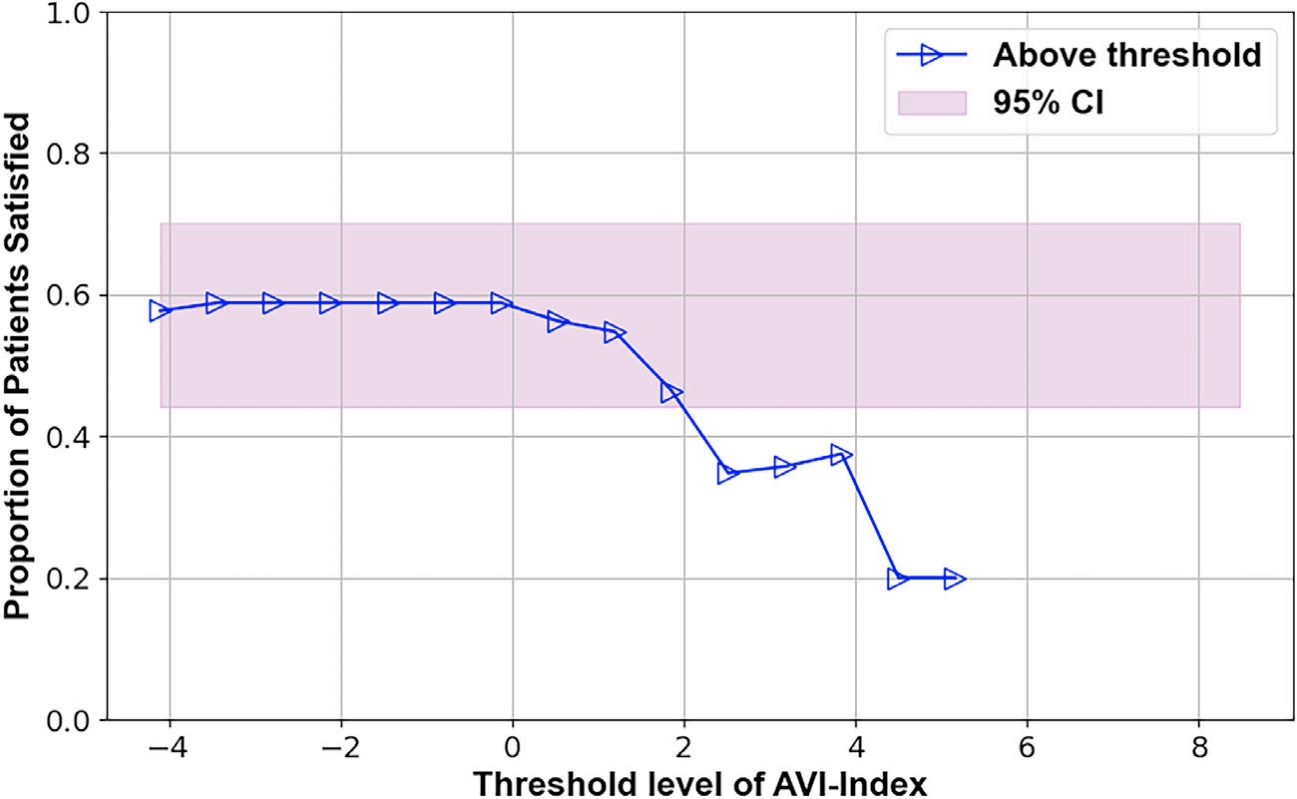
TL graph indicating the potential of the anterior vertical instability index to predict patient satisfaction 1 year following decompression-only surgery for degenerative spondylolisthesis.

**Fig. 5:**
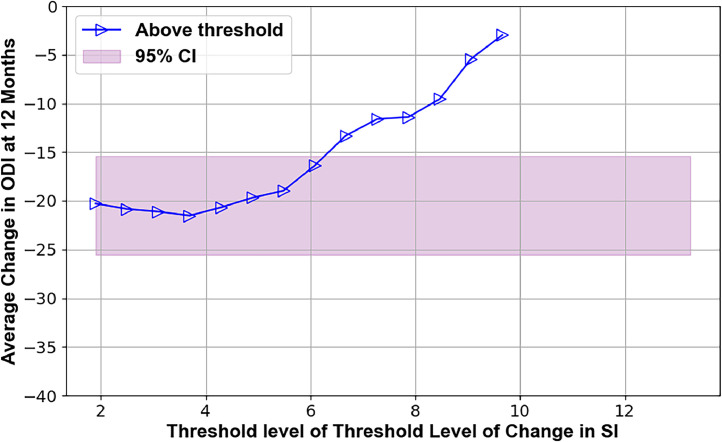
TL graph indicating the potential of the change in the SI in flexion versus extension to predict the mean improvement in ODI scores at 1 year following decompression-only surgery.

**Fig. 6:**
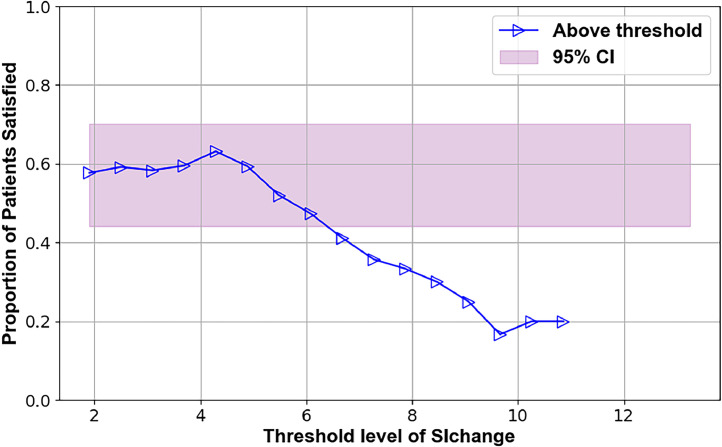
TL graph indicating the potential of the change in the SI in flexion versus extension to predict patient satisfaction 1 year after decompression-only surgery.

**Fig. 7:**
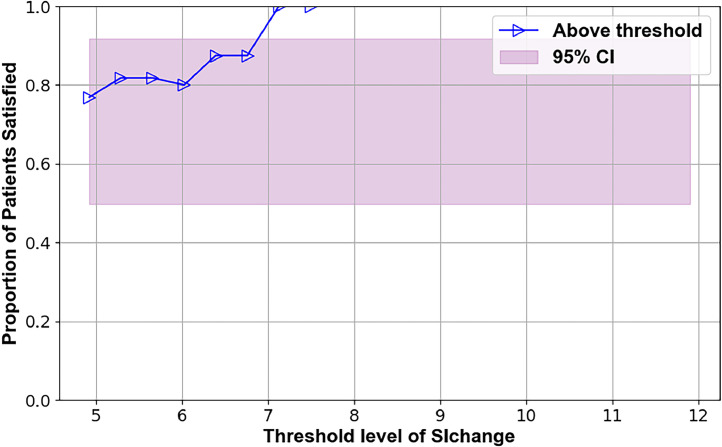
TL graph indicating the potential of the change in the SI in flexion versus extension to predict the proportion of satisfied patients 1 year after decompression plus fusion surgery.
• Translational or vertical instability at a specific level may be only 1 of several factors contributing to lumbar spinal symptoms. Confounding issues, such as varying degrees of intervertebral disc and facet degeneration, endplate changes across multiple levels, vacuum signs in the discs or facet joints, fatty substitution in muscles, sacroiliac joint degeneration, and psychosocial factors, might also influence outcomes [[31], [32], [33], [34], [35], [36], [37]]. These complex, multifactorial elements make it challenging to isolate the impact of a single preoperative intervertebral motion abnormality on patient-reported outcomes following surgery. Randomized controlled trials with stratification, matched cohort studies, multivariable predictive modeling, or other sophisticated study designs are required to better understand how a translational or vertical instability diagnosis might help optimize patient outcomes.
• In the retrospectively analyzed prospective study, translational instability was used to help determine whether fusion should be added to decompression. The index used in that study was the Sagittal Plane Instability Index (SPSI), which is linearly related to the TI-Index [7]. In the prospective study, if the translational instability was > 2, fusion was generally used in addition to decompression [13]. Thus, retrospective analysis of the data to investigate the TI-Index's potential to predict outcomes is limited by its use in treatment selection and is therefore not emphasized in our study.
• Only 12-month outcomes were analyzed since there were fewer 24-month outcomes data. However, the 12-month outcomes have been shown to be predictive of longer-term outcomes [[38], [39], [40]]. The patient reported outcomes may vary with preoperative counseling and individual expectations [41]. A much larger sample, with collection and analysis of confounding variables, is needed to understand the relative importance of intervertebral motion metrics fully.
• The findings may not be generalizable to surgical decompression and fusion techniques other than used in the prospective study. Different decompression methods can have varying effects on segmental stability [14,[42], [43], [44], [45]]. These differences may interact with factors such as facet angle, disc height loss, and pre-existing instability. Likewise, variations in fusion techniques can also influence the outcomes [46].
• Good patient effort is essential to obtain reliable measurements from flexion-extension radiographs [7]. The prospective study used a standardized flexion-extension protocol to obtain the radiographs we analyzed [13]. A technician training video (https://www.youtube.com/watch?v=YDcMMZdc7dc) and patient education videos in English (https://youtu.be/KaEQP57qWgU) and Dutch (https://www.youtube.com/watch?v=XZyKkv3zO20) are available to teach this protocol. At least 5° of rotation was found at all treatment levels analyzed. This amount of rotation has been hypothesized to be sufficient for the reliable diagnosis of intervertebral motion abnormalities [7]. However, this hypothesis has yet to be rigorously tested.

Several preoperative metrics were identified using the TL plots that may predict 12-month outcomes. These candidate predictors are illustrated in Figs. 2–7, with supporting statistics and detailed observations in Table 1. In particular, the AVI-Index may be predictive of 1 year outcomes in decompression-only patients (Fig. 2). The change in ODI score at 12 months was significantly less (-11±12) for patients where the preoperative treatment level AVI-Index was >3 compared to patients where the AVI-Index was ≤3 (-24±19), p=.016. The AVI-Index may also be predictive of patient satisfaction after decompression-only surgery (Fig. 4). The proportion of satisfied patients at 12 months was significantly less (0.31 std err 0.12) for patients where the preoperative treatment level AVI-Index was > 3 compared to patients where the AVI-Index was ≤ 3 (0.69 std err 0.076), p=.005. The change in the SI in flexion versus extension may predict the proportion of satisfied patients 1 year after decompression plus fusion surgery (Fig. 7). The proportion of satisfied patients was higher (1 versus 0.57, p=.034) when the SI changed by over 7 between flexion and extension preoperatively.

## Discussion

Deciding whether to perform decompression alone or add fusion in patients with lumbar stenosis and spondylolisthesis remains a common and unresolved clinical challenge. Given the inconclusive evidence guiding this decision, we retrospectively analyzed prospectively collected data to explore whether specific preoperative radiographic metrics could help improve surgical decision-making.

The TL-graphs in Fig. 1, Fig. 2, Fig. 3, Fig. 4, Fig. 5, Fig. 6 and the additional statistical analyses summarized in Table 1 suggest criteria for intervertebral motion and alignment metrics that might be tested in subsequent clinical trials. The proposed criteria used are outlined in Table 2. The threshold levels suggested in Table 2 must be tested and likely refined in a much larger study. The data in Fig. 1, Fig. 2, Fig. 3 indicate that vertical instability, measured using the AVI-Index, may predict 12-month outcomes following decompression-only surgery. As the preoperative AVI-Index increased into the abnormal range, patients showed smaller improvements in ODI scores and leg pain, and fewer were satisfied at 1 year. These findings suggest that decompression alone may not adequately address issues caused by vertical instability. In contrast, among patients who underwent decompression plus fusion, the preoperative AVI-Index did not appear to affect the outcomes.

**Table 1:** Summary of threshold limit graphical analysis findings and subsequent statistical tests of the preoperative intervertebral motion metrics that may affect patient outcomes following decompression alone or decompression plus fusion surgery for lumbar spinal stenosis with spondylolisthesis

Imaging metric	Patient outcome	Suggested threshold	Observation
**Decompression only surgery**			
AVI-index Fig. 1	Change in ODI at 12 months	3	Change in ODI score at 12 months was significantly less (-11±12) for patients where the preoperative treatment level AVI-Index was >3 compared to patients where the AVI-Index was ≤3 (-24±19), p=.016
AVI-index Fig. 2	Change in NRS leg pain at 12 months	3	A trend (p=.06) toward the change in pain score at 12 months being less (−2.2±2.5) for patients where the preoperative treatment level AVI-Index was >3 compared to patients where the AVI-Index was ≤3 (−4.1±3.3)
AVI-index Fig. 3	Proportion of satisfied patients at 12 months	3	Proportion of satisfied patients at 12 months was significantly less (0.31 std err 0.12) for patients where the preoperative treatment level AVI-Index was >3 compared to patients where the AVI-Index was ≤3 (0.69 std err 0.076), p=.005
Change in SI, Flexion-to-Extension Fig. 4	Change in ODI at 12 months	8	A trend (p=.08) for worse outcomes (11±16 vs. 23±18 point improvement) if the change in the SI between flexion and extension was <8 preoperatively.
Change in SI, Flexion-to-Extension Fig. 5	Proportion of satisfied patients at 12 months	8	Proportion of satisfied patients is significantly lower (0.33 versus 0.68, p=.049) for patients where the change in the SI between flexion and extension was >8.
**Decompression plus fusion surgery**			
Change in SI, Flexion-to-Extension Fig. 6	Proportion of satisfied patients at 12 months	7	The proportion of satisfied patients was higher (1 vs 0.57, p=.034) when the SI changed by over 7 between flexion and extension preoperatively.

In the observation column, the statistics are based on 1-way analysis of variance or a test of proportions (Stata ver 15, Stata Corp). Note that the TL graphs plot averages or proportions above a threshold level, while the analysis of variance and proportion tests compare data based on whether the data are above or below a threshold. Thus, there are some apparent differences between the observations made from the TL graphs and the results of the statistical testing. The TL graphs were only used to help identify thresholds for subsequent statistical tests. Also note that a point is plotted on the TL-graphs only if there were at least 4 data points available to calculate the average or proportion. This also results in some apparent discrepancies between the TL-Graphs and the results of the statistical tests.

**Table 2:** Summary of preoperative radiographic metrics that, if validated in subsequent studies, may prove effective in treatment algorithms to choose between decompression only versus decompression plus fusion surgery for patients with lumbar spinal stenosis and spondylolisthesis

Preoperative radiographic Metric	Potential to predict outcomes following	
	Decompression only	Decompression plus fusion
AVI-index	Outcomes worse if >3	No association
Change in SI between flexion and extension	Outcomes worse if >8	Outcomes better if >7

The data in Figs. 4 and 5 suggest that the change in the SI between flexion and extension may predict the outcome of decompression surgery. With a large change in the SI, lesser improvements in ODI were found, and fewer patients were satisfied at 1 year. It has previously been documented that not all lumbar spondylolistheses are unstable [[25], [26], [27], [28]]. However, the criteria for differentiating between stable and unstable spondylolisthesis must be rigorously validated. With further validation, the SI could prove effective.

One year after decompression plus fusion, the satisfaction rates were higher when the preoperative SI change between flexion and extension was high (Fig. 6). Although the small sample size limits the interpretation, this finding is consistent with the idea that fusion may be more beneficial for dynamic spondylolisthesis. However, spondylolisthesis and instability are influenced by multiple factors, which makes these relationships complex [29,30]. Both the change in SI between flexion and extension and the TI-Index are measures of translational instability, though they quantify different aspects of translation [[7], [18]]. While they were somewhat correlated in this study (p<.0001), the low R² of 0.2 suggests they capture different aspects of instability and may have unique predictive value. While no single metric can perfectly predict outcomes, they may help estimate success probabilities. For example, although all 7 patients with a preoperative SI change of >7 who underwent decompression plus fusion were satisfied at 12 months, 6 of 13 patients with a similar SI change treated by decompression alone were also satisfied at 12 months.

These findings should only be considered as clues to what preoperative metrics might help to predict surgical outcomes. However, it is particularly encouraging when TL graphs with different outcome measures (eg, disability, pain, and satisfaction, as illustrated in Fig. 1, Fig. 2, Fig. 3) all support a particular preoperative metric's predictive capacity and threshold level. Further research is required in this regard. If these findings can be repeated in additional clinical trials, they would support that multiple standardized biomechanical metrics derived from radiographs should be considered to achieve optimal surgical treatment outcomes (as opposed to a single translational instability metric as used in many prior research studies).

### Limitations

• The primary limitations are the small sample size and inherent variability in outcomes in this patient population. This variability is indicated by the width of the pink-shaded regions in Fig. 2, Fig. 3, Fig. 4, Fig. 5, Fig. 6, Fig. 7. The shaded areas represent 95% confidence intervals for the outcome data. As the sample size increases, the width of the shaded region decreases, making it easier to detect significant differences.

Many authors have called for improved approaches to treat lumbar spinal stenosis [[47], [48], [49], [50], [51]]. Optimizing treatment likely requires integrating multiple clinical and imaging factors tailored to each patient [[52], [53], [54], [55]]. Validated criteria are needed to determine which patients may benefit most from adding fusion to decompression surgery [1,2,51,56]. Because numerous variables influence outcomes, large datasets are essential to develop and validate diagnostic and treatment algorithms [26,[57], [58], [59], [60], [61], [62], [63], [64], [65]]. These algorithms should identify the most predictive preoperative variables, guide surgical decisions, and clarify when fusion offers better outcomes and cost-effectiveness than decompression alone [66].

The current study is exploratory in nature. Much larger clinical studies are needed with the statistical power to account for all critical variables to develop highly effective diagnosis and treatment algorithms. However, evidence is required to select the factors to include in large-scale studies. Spinal stability is 1 factor that might be included, considering that most surgeons consider stability important in treatment selection [3]. Simple translation or rotation measurements could be used to assess for instability, though they have multiple limitations, and there is no reliable evidence that these simple measurements are effective [7]. We have demonstrated that several more advanced spinal stability metrics can be automatically measured from preoperative flexion-extension radiographs. These metrics address the limitations of using translation or rotation to assess for instability [7]. The threshold-limit graphical approach may help identify promising spinal stability metrics and their associated threshold levels that could prove clinically effective in determining the optimal surgery. Once these metrics and thresholds have been identified, they can be rigorously tested in large clinical trials using conventional statistical methods and more sophisticated, multifactor modeling, potentially leading to more personalized and evidence-based treatment strategies.

## Conclusion

This retrospective analysis of prospectively collected data identified measurements made from preoperative lumbar flexion-extension radiographs that may help determine whether a patient with lumbar spinal stenosis with spondylolisthesis will benefit from fusion in addition to decompression surgery. The results are hypothesis-generating and align with the field’s push toward personalized treatment selection algorithms. Our preliminary findings suggest that vertical instability (eg AVI-Index >3) and dynamic spondylolisthesis (eg a change in the SI-Index >7) should be considered in assessing spinal stability, in addition to abnormal translational instability (eg TI-Index >2). Comprehensive, multicenter clinical trials with a much larger sample are needed to develop and validate threshold levels of each metric, along with diagnosis and treatment algorithms to optimize outcomes for patients with lumbar stenosis and spondylolisthesis.

During the preparation of this work the author(s) used ChatGPT-o4 and GROK-3 in order to provide a “peer” review of the manuscript, identify areas that lack clarity, and suggest improvements in writing. After using this tool/service, the author(s) reviewed and edited the content as needed and take(s) full responsibility for the content of the publication.

## Declaration of competing interest

The authors declare the following financial interests/personal relationships which may be considered as potential competing interests: John A. Hipp and Trevor F. Grieco are employees of Medical Metrics, Inc and own stock in Medical Metrics, Inc. Job Van Susante previously was awarded a research grant from Medical Metrics, Inc. Bradford Currier is a paid consultant for Medical Metrics, Inc.

One or more authors declare potential competing financial interests or personal relationships as specified on required ICMJE-NASSJ Disclosure Forms.

## Appendix Supplementary materials

Image, application 1
