# Supplementary material for: Identifying preoperative radiographic metrics to guide surgical selection in lumbar spondylolisthesis and stenosis
Source: N Am Spine Soc J. 2025 Aug 21;24:100784. doi: 10.1016/j.xnsj.2025.100784 (PMC12493226; doi:10.1016/j.xnsj.2025.100784)
Supplement: Supplementary file 1 [file mmc1.docx]

Appendix 1: Input parameters used to produce Threshold-Limit graphs. These parameters were used for both decompression-only and decompression-plus fusion patients. All data were filtered to include only the index-level data.

| Outcome to be predicted | Preop metric tested | Filter |
| --- | --- | --- |
| Change in ODI @ 12 m | Translational instability index | Rotation > 3 |
| Change in ODI @ 12 m | Vertical instability index | Rotation > 3 |
| Change in ODI @ 12 m | Change in Spondylolisthesis index between flexion-extension |  |
| Change in ODI @ 12 m | Disc height index |  |
| Change in NRS @ 12 m | Translational instability index | Rotation > 3 |
| Change in NRS-LP @ 12 m | Vertical instability index | Rotation > 3 |
| Change in NRS-LP @ 12 m | Change in Spondylolisthesis index  between flexion-extension |  |
| Change in NRS-LP @ 12 m | Disc height index |  |
| Proportion of patients where 12 m ODI improved by >= 20 | Translational instability index | Rotation > 3 |
| Proportion of patients where 12 m ODI improved by >= 20 | Vertical instability index | Rotation > 3 |
| Proportion of patients where 12 m ODI improved by >= 20 | Change in Spondylolisthesis index between flexion-extension |  |
| Proportion of patients where 12 m ODI improved by >= 20 | Disc height index |  |
| Proportion of patients satisfied @ 12 m | Translational instability index | Rotation > 3 |
| Proportion of patients satisfied @ 12 m | Vertical instability index | Rotation > 3 |
| Proportion of patients satisfied @ 12 m | Change in Spondylolisthesis index between flexion-extension |  |
| Proportion of patients satisfied @ 12 m | Disc height index |  |
